# The impact of Longitudinal Integrated Foundation Training: preliminary experiences in the United Kingdom

**DOI:** 10.1186/s12909-023-04850-9

**Published:** 2023-11-16

**Authors:** Kathryn E. Burnett, Suzanne Gawne, James Barrett, David Baxter, Catherine Tregoning, Paul Baker

**Affiliations:** 1 NHS Organisational Consultancy; Hosted Business Services (SH0142), Salford Royal NHS Foundation Trust, Part of the Northern Care Alliance, Hope Building, Stott Lane, Salford, M6 8HD UK; 2 Medical Directors Office, East Lancashire Hospitals NHS Trust, Blackburn, UK; 3 Wirral University Teaching Hospital, Wirral, UK; 4 Stockport NHS Foundation Trust, Stockport, UK; 5 Chartered Psychologist, Cheshire, UK; 6 Workforce Training & Education Directorate, NHS England North West, Liverpool, UK

**Keywords:** Doctors, Foundation programme, Longitudinal Integrated Foundation Training, General practice

## Abstract

**Background:**

The United Kingdom health system is challenged with retaining doctors entering specialty training directly after their second foundation year. Improving doctors’ training experience during the foundation programme may aid such retention. The Longitudinal Integrated Foundation Training (LIFT) pilot scheme aimed to provide a programme that improves the quality of their foundation training experience, advance patient-centred care and provide doctors with more experience in the primary care settings.

**Methods:**

During this pilot study, three methods were employed to evaluate and compare doctors’ experiences across their 2-year foundation training programme: Horus ePortfolio assessment of six domains for good medical practice analysed using a T-test, online survey assessments analysed using a 2-tailed chi-square test, and focus group feedback sessions with thematic analysis.

**Results:**

Doctors completing LIFT (n = 47) scored a higher but non-significant mean score on all six domains for good medical practice *versus* doctors completing traditional foundation training (n = 94). By the end of foundation training, 100% of LIFT doctors rated their understanding of how primary and secondary care work together as high *versus* 78.7% of traditional doctors (p < 0.05). Improvements in wellbeing were observed among LIFT doctors, along with a reduction in the proportion of doctors considering leaving medical training. A significantly greater number of LIFT doctors *versus* traditional doctors rated their compassion for patients as high (100% *versus* 86.8%; p < 0.05), intended to become general practitioners (23.1% *versus* 13.5%; p < 0.05) and rated the extent to which they felt well informed and able to consider a general practice career rather than a hospital career as high (91.7% *versus* 72.3%, respectively; p < 0.05). Some LIFT doctors felt they had reduced exposure to secondary care, received less on-call experience and considered working a half-day to be problematic; challenges ameliorated by the end of the 2-year foundation programme.

**Conclusion:**

The LIFT programme enhanced the quality of foundation training and improved doctors’ experiences and competencies, generating valuable insights for the future of education and healthcare delivery. Applying the principles of LIFT to foundation training helps doctors to be more compassionate and patient-centred, leading to enhanced individualised patient care.

## Background

The current foundation programme (FP) in the United Kingdom (UK) is a work-based, 2-year postgraduate training programme that newly qualified doctors complete following their undergraduate medical training [1, 2]. These doctors typically experience six 4-month clinical placements rotating through different specialties. Of these, five are in the secondary care setting (hospital) and one is in the community setting (e.g. general practice [GP]) [2, 3]. Following this 2-year foundation training programme, doctors then choose which specialty they wish to pursue in their future careers. Of concern is the steady decline in the number of doctors entering specialty training after their Foundation Year 2 (F2), falling from 71.3% in 2011 [4] to 37.7% in 2018 [5, 6]. Similarly, there has been a steady increase in the number of doctors reporting taking a career break, from 4.6% in 2011 to 13.6% in 2019 [6]. Although news reports and articles are highlighting the increasing number of UK doctors taking time out of training [7], there are few peer-reviewed, large-scale qualitative research studies that explore this changing culture. The growing shortage of general practitioners in the UK [8] may be partly as a consequence of foundation doctors gaining experience of the community setting later on in their foundation training after they have made a decision about their career choice [3]. This, in combination with the increasing shortage of doctors, demonstrates potential limitations in the current FP and, despite the existence of programmes to help support and retain UK doctors (e.g. Foundation Priority Programme) [9] and increasing opportunities for flexible working and/or reduced hours, there may be a need to reconsider alternatives to the traditional training model.

Organisational change can be beneficial, and introducing novel approaches to traditional methods of medical education could support relationship continuity between doctors and patients and improve the doctors’ experience during clinical training. Longitudinal programmes incorporate a continuous method of training in particular settings rather than in the traditional block sessions, and there has been much research into their effects on undergraduate medical students. The advantage of innovative longitudinal programmes in medical schools across Australia, Canada, South Africa and the United States was the opportunity for undergraduate students to view individual patients with multiple medical problems via a more holistic approach, with a higher proportion of students entering the primary care setting than those on traditional training programmes [10]. Other models have shown that students have a greater sense of purpose in their education and perform higher academically than their traditionally trained peers [11]. Longitudinal training has also proved successful at Harvard Medical School when applying the Cambridge Integrated Clerkship model, which fosters students’ learning to advance their professional development. Students demonstrated clinical skills that were on par with or better than those on the traditional training programme, more confidence in the domains of patient care and a stronger sense of patient-centred care [12]. Much less is known, however, about the impact of longitudinal programmes at the postgraduate level.

Considering the positive impact that implementation of longitudinal training programmes has had on the undergraduate student experience in other countries, and following the example of Harvard Medical School [12], Longitudinal Integrated Foundation Training (LIFT) was introduced by the North West of England Foundation School (NWoEFS) in August 2016 [13]. In this pilot scheme, postgraduate doctors experienced three sessions per week in the same primary care setting throughout their 2 years of foundation training, alongside five sessions each week in a hospital placement; each hospital placement lasted for 4 months.

The LIFT programme was undertaken across eight FPs in the NWoEFSs – in Bolton, East Lancashire, Morecambe Bay, Pennine Acute, South Manchester, Stockport, Wigan and Wirral. The scheme aimed to provide doctors with more experience in the primary care setting and improve the quality of their foundation training experience while also supporting the development of patient-centred and holistic care. The success of this scheme was assessed by comparing outcomes with those of doctors on the traditional FP. The scheme also provided a unique opportunity to assess whether foundation training was enhanced through the LIFT model in line with the Department of Health mandate [14] where the objectives are, in part, to improve the quality of education and training of doctors, thus supporting the National Health Service (NHS) in delivering world-class compassionate care.

## Methods

This research was approved by Health Education England research governance. Across the 8 trusts sites, doctors applying for foundation posts were matched to their highest choice based on a meritocratic algorithm, and so not all doctors selected the LIFT programme despite being enrolled.

A variety of methods were employed to determine doctors’ experiences and perceptions during this pilot study (foundation training period 2016 to 2018) and, to ensure impartiality, an external, independent consultant, with a background in health psychology, conducted the research and analysis of the results.

### Horus electronic portfolio (ePortfolio) evaluation

Horus is the ePortfolio system on which all foundation doctors in England are required to record and reflect on career planning and their educational and professional development. ePortfolios were evaluated blindly by two independent researchers after F2. As the number of traditional doctors exceeded the number of LIFT doctors, a 1:2 sampling method (one ePortfolio from the LIFT group being analysed alongside two from the traditional group) was employed. The General Medical Councils’ six domains for good medical practice were mapped onto corresponding themes from the NHS constitution and also the LIFT project aims. The resulting six domains (Domain 1: Patient safety; Domain 2: Patient-centredness; Domain 3: Quality improvement; Domain 4: Self-regulation and personal development; Domain 5: NHS values – compassion; Domain 6: Leadership and management) were then used as a framework against which to analyse the data from the ePortfolios. An ordinal scale (0–2) was used to code ePortfolio documents to show whether doctors showed a considerable amount of evidence (2) to support each domain, some evidence (1) or no evidence at all (0).

### Survey assessment

As part of the LIFT programme, an online survey was conducted; 44 LIFT doctors, 1684 traditional doctors and 474 supervisors were invited to take part within the participating NWoEFSs to evaluate perceptions. Some supervisors were involved in the supervision of both LIFT and traditional doctors. The survey was conducted at the end of Foundation Year 1 (F1) and F2 and was designed specifically for each of the foundation doctor and supervisor groups. The questions asked were kept consistent each year in order to maintain and assess the longitudinal effect, with the same groups asked to contribute to the survey in both F1 and F2. An email invitation containing a link to the online survey was distributed to all participants, accompanied by an information sheet and consent form. Where possible on each occasion, doctors and their supervisors were asked similar questions, thus allowing comparative data to be gained. Survey ratings for the various parameters were given out of 10; ≥7 out of 10 was classified as a high rating.

### Focus group analysis

A first phase of eight focus groups took place (one at each of the participating NWoEFSs) and all 47 LIFT doctors were invited to participate between January to April 2017 (during F1) to assess doctors’ perceptions of LIFT across all eight sites; they lasted up to 1 h 49 min. A semi-structured interview technique was applied to explore areas of most interest and relevance to the doctors themselves. These eight focus group sessions were repeated during a second phase between March and June 2018 (during F2) with all 47 LIFT doctors again invited to participate, and the data compared with the first phase to determine changes in perceptions. Traditional doctors were not involved in the focus groups. Participants answered eight questions in F1 and an extra question was added to the focus group in F2; the original eight questions related to doctors’ experiences, progress, relationships with their patients/supervisors/teams and the potential impact of LIFT upon their career decisions. The extra question added to the focus group in F2 pertained to any developments or changes that may have been made to the provision of LIFT as the programme progressed. Where possible, the questions sought to replicate those being asked in parallel of LIFT supervisors during telephone interviews conducted by another member of the research team, in order to obtain comparative data. Each focus group session was recorded, transcribed verbatim and the central themes identified.

To add validity to the responses, a second analysis was conducted by an additional researcher independent to the first. This took place upon completion of focus groups in F1 and F2. In each case, half of the focus groups that took place were re-analysed. Due to repetition across the groups, it was decided that half of the data would suffice as a representative sample. Consequently, the results of the second analysis of the 2018 Phase 2 groups are presented here.

### Analyses

ePortfolios were analysed from both LIFT and traditional doctors and mean scores were calculated for each of the six domains for good medical practice. The size difference between the F2 LIFT and traditional doctor groups was analysed using a T-test, conducted on the resulting mean scores attributed for the six domains.

Assessment of survey data was carried out using a 2-tailed chi-square test, where appropriate, and p < 0.05 was considered statistically significant.

Focus group sessions were transcribed and a thematic analysis was undertaken.

### Reflexivity statement

KEB and PB are educational leaders with a vision to explore and evolve the way medical education is delivered. As such, they are inclined towards the success of the scheme. SG, JB and DB are directors of medical education (DMEs) and are responsible for ensuring the ongoing education and training of doctors while also maintaining patient safety. For this assessment, organisations were invited to apply to be a pilot site, and so by definition the DME authors are early adopters; however, they are regarded as unbiased. Indeed, the success of the pilot is not reflective of their performance and, if it was felt that the pilot was not meeting its intended outcomes, the DMEs would have had a responsibility to highlight this throughout the scheme for the purposes of maintaining patient safety. CT is an external, independent consultant, with a background in health psychology, and conducted the study and analysed the results to ensure impartiality.

## Results

### Horus ePortfolio evaluation

**Fig. 1:**
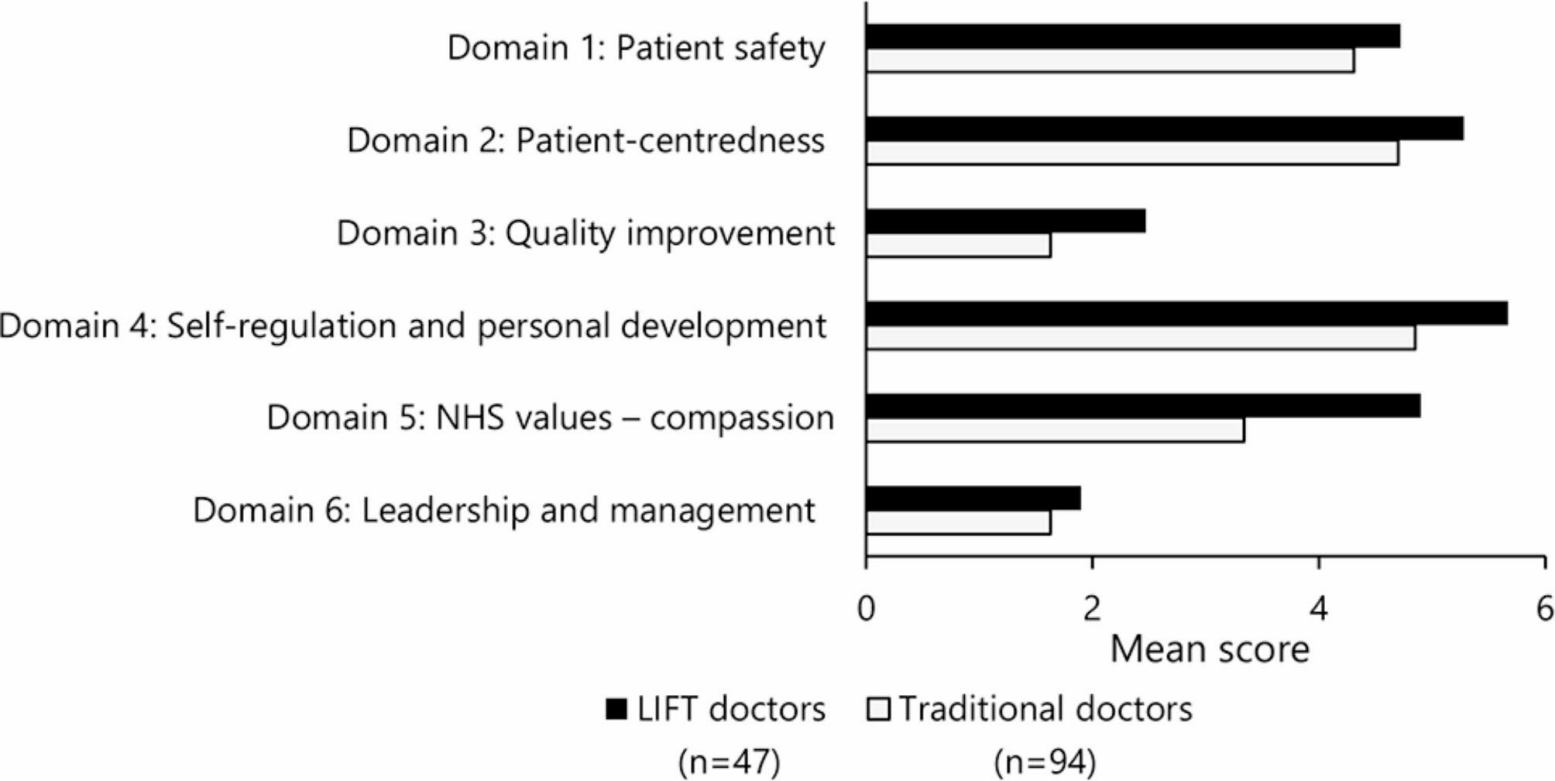
LIFT *versus* traditional doctors’ mean scores for each domain for good medical practice in F2 F2, Foundation Year 2; LIFT, Longitudinal Integrated Foundation Training; NHS, National Health Service

A total of 141 doctor ePortfolios were evaluated: the complete cohort of LIFT doctors (n = 47), together with a random selection of ePortfolios for traditional doctors (n = 94). The LIFT doctors consistently scored a higher mean score on all six domains for good medical practice than traditional doctors (differences were not statistically significant; Fig. 1). The largest difference between the mean scores of the two groups was 1.56 and observed in relation to NHS values – compassion, suggesting that the LIFT programme made the most difference to doctors in this domain.

### Survey assessments

At the end of F2, 12 LIFT doctors, 408 traditional doctors and 202 supervisors responded to the online survey (response rates of 27.3%, 24.2% and 42.6%, respectively). This report focuses on LIFT doctors and their experiences; however, some supervisor data are also provided below for context.

#### Perception of doctors’ experience

**Fig. 2:**
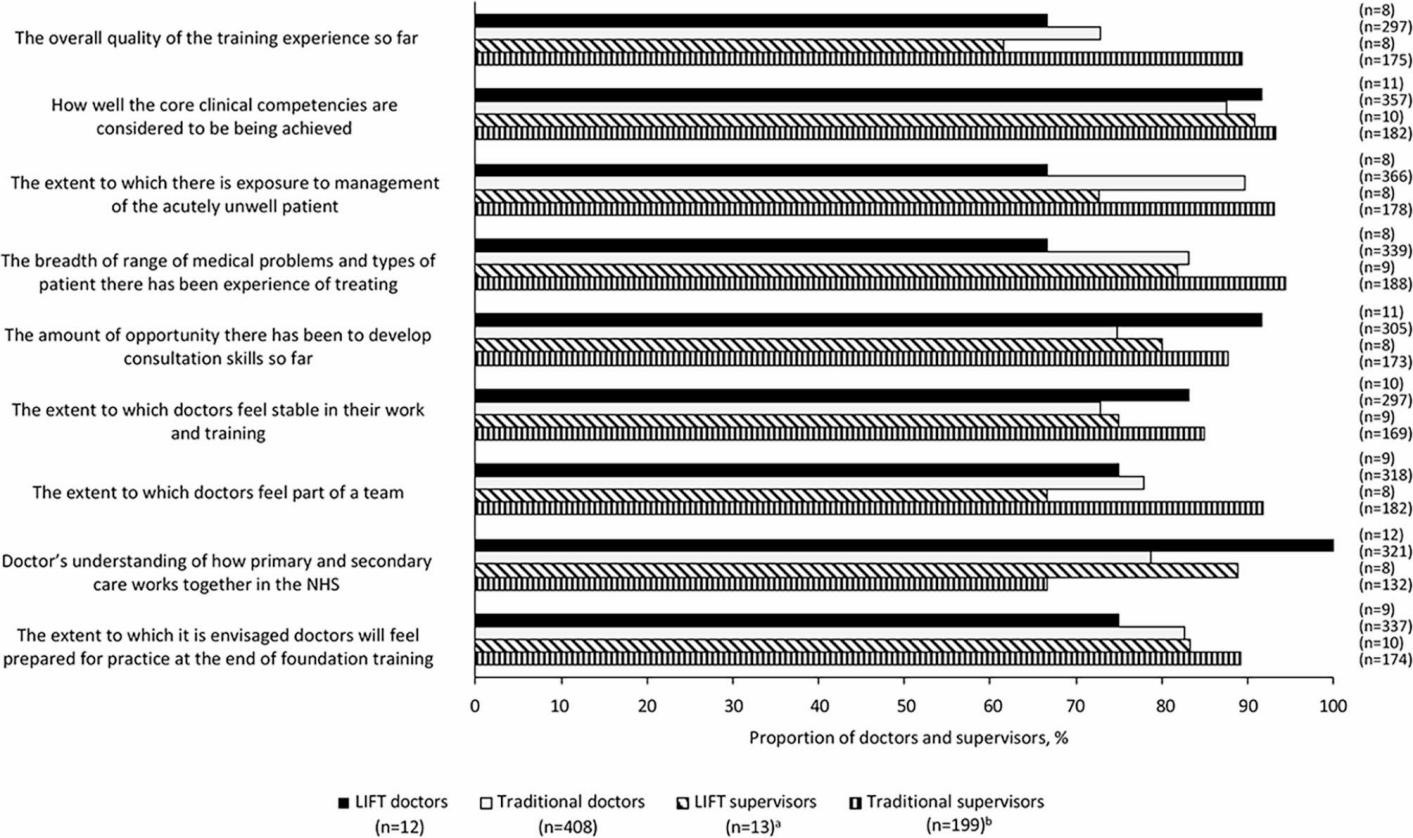
Proportion of LIFT doctors, traditional doctors and their supervisors who rated their experiences of foundation training as good or high in F2 ^a^The number of LIFT supervisors responding ranged from 9 to 13 ^b^The number of traditional supervisors responding ranged from 191 to 199 F2, Foundation Year 2; LIFT, Longitudinal Integrated Foundation Training; NHS, National Health Service

By the end of the FP, significantly more LIFT than traditional doctors reported that they had felt stable in their work and training (i.e. considered themselves to be more settled and not having missed out on their medical training) to a high extent (83.3% *versus* 72.8%, respectively; p < 0.05; Fig. 2). Also significant was that 100% of LIFT doctors rated their understanding of how primary and secondary care work together in the NHS as high, compared with 78.7% of doctors on the traditional FP (p < 0.05). More LIFT doctors than traditional doctors also considered that they had a greater opportunity to develop consultation skills. The traditional FP was perceived by both supervisors and doctors as providing better exposure to management of the acute unwell patient and a better experience of treating a breadth of range of medical problems and types of patients than the LIFT programme.

#### Wellbeing of doctors

**Fig. 3:**
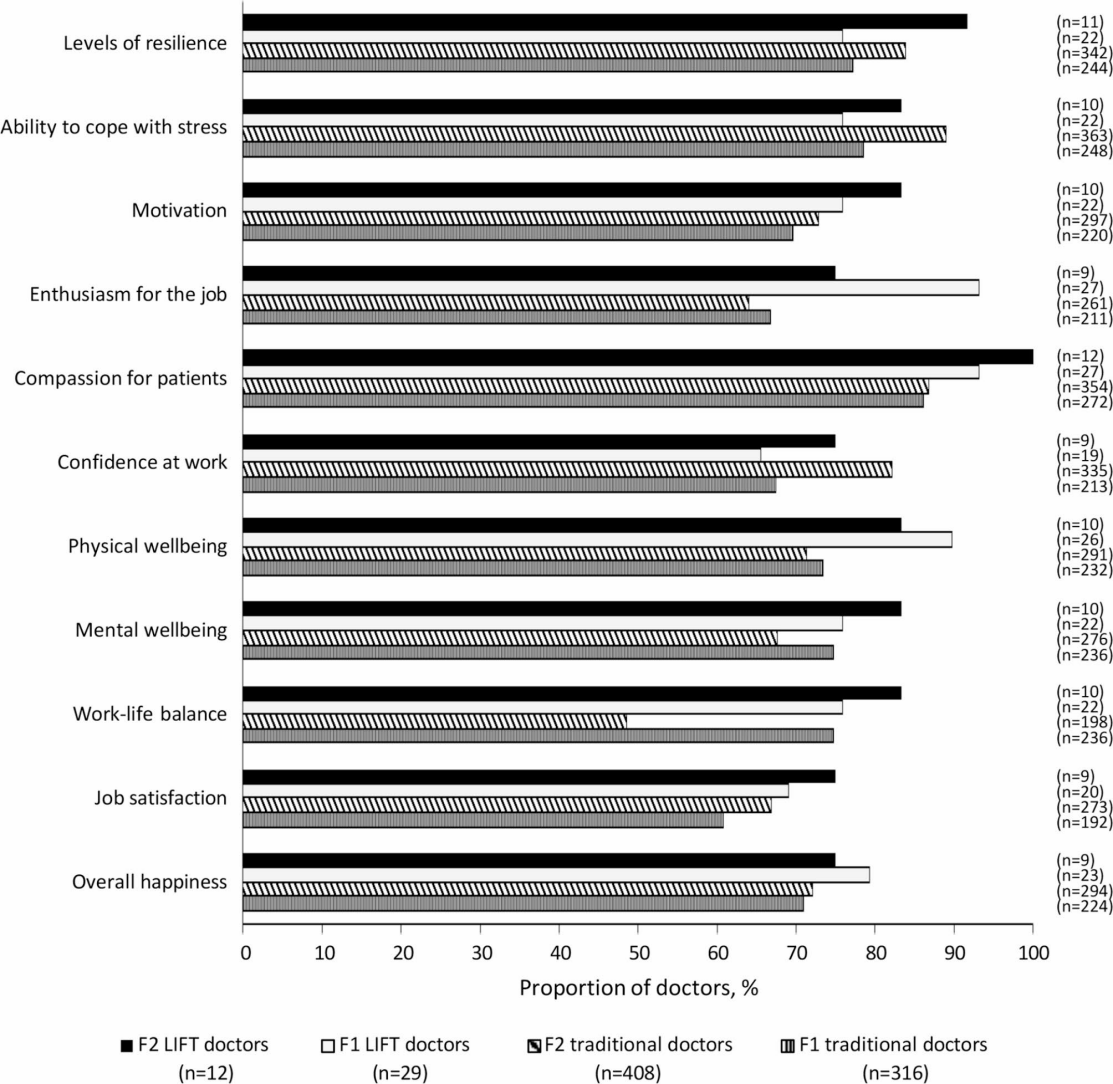
Proportion of LIFT and traditional doctors rating themselves as high for parameters of wellbeing, across F1 and F2 F1, Foundation Year 1; F2, Foundation Year 2; LIFT, Longitudinal Integrated Foundation Training

Self-perceptions of wellbeing among LIFT doctors improved in F2 on 9 of 11 measures, with the exceptions of physical wellbeing and overall happiness. By contrast, self-perceptions of wellbeing among traditional doctors improved on only 7 of 11 measures (Fig. 3). The number of LIFT doctors rating themselves as highly resilient also significantly increased over F2, from 75.9% to 91.7% (p < 0.05), compared with an increase from 77.2% to 83.8% for traditional doctors. Significantly, 100% of LIFT doctors rated their compassion for patients as high compared with 86.8% of traditional doctors (p < 0.05).

An overall improvement in feelings of depression was generally observed among LIFT doctors when compared with traditional doctors, as well as a general reduction in their frequency of thoughts of leaving the medical training pathway (Table 1). The numbers of LIFT doctors who never or very rarely felt depressed significantly improved, from 75.9% in F1 to 91.7% in F2 (p < 0.05), compared with 78.5% in F1 to 69.0% in F2 of traditional doctors. Moreover, the numbers of LIFT doctors who never or very rarely thought about leaving their training pathway also significantly improved from 51.7% in F1 to 83.3% in F2 (p < 0.05), compared with 68.4% in F1 to 63.7% in F2 of traditional doctors. By the end of foundation training, significantly fewer LIFT doctors (8.3%) remained feeling stressed most or all of the time, compared with 19.9% of traditional doctors (p < 0.05).

**Table 1:** Proportion of LIFT and traditional doctors experiencing parameters of wellbeing across F1 and F2

Parameter	Doctor type	Year (n)	Proportion of doctors, % (n)					
			Never	Very rarely	Sometimes	Several times each day	Most of the time	All of the time
Stress	LIFT doctors	F2 (12)	0.0 (0)	16.7 (2)	**41.7** (**5**)	**33.3** (**4**)	0.0 (0)	8.3 (1)
		F1 (29)	0.0 (0)	10.3 (3)	**62.1** (**18**)	**13.8** (**4**)	10.3 (3)	3.4 (1)
	Traditional doctors	F2 (407)	2.2 (9)	9.6 (39)	**48.9** **(199)**	**19.4** (**79**)	16.2 (66)	3.7 (15)
		F1 (316)	0.0 (0)	11.4 (36)	**45.6** **(144)**	**26.6** (**84**)	15.2 (48)	1.3 (4)
Anxiety	LIFT doctors	F2 (12)	0.0 (0)	**33.3** (**4**)	**41.7** (**5**)	16.7 (2)	0.0 (0)	8.3 (1)
		F1 (29)	0.0 (0)	**34.5** (**10**)	**37.9** (**11**)	13.8 (4)	10.3 (3)	3.4 (1)
	Traditional doctors	F2 (407)	5.2 (21)	**23.8** (**97**)	**48.2** **(196)**	10.3 (42)	10.3 (42)	2.2 (9)
		F1 (316)	8.9 (28)	**16.5** (**52**)	**53.2** **(168)**	13.9 (44)	7.6 (24)	0.0 (0)
Feelings of depression	LIFT doctors	F2 (12)	**16.7** (**2**)	**75.0** (**9**)	0.0 (0)	0.0 (0)	0.0 (0)	8.3 (1)
		F1 (29)	**24.1** (**7**)	**51.7** (**15**)	20.7 (6)	3.4 (1)	0.0 (0)	0.0 (0)
	Traditional doctors	F2 (407)	**29.0** **(118)**	**40.0** **(163)**	25.8 (105)	1.5 (6)	2.9 (12)	0.7 (3)
		F1 (316)	**31.6** **(100)**	**46.8** **(148)**	15.2 (48)	5.1 (16)	1.3 (4)	0.0 (0)
Thoughts of leaving the medical training pathway	LIFT doctors	F2 (12)	**58.3** (**7**)	**25.0** (**3**)	8.3 (1)	0.0 (0)	0.0 (0)	8.3 (1)
		F1 (29)	20.7 (6)	**31.0** (**9**)	**24.1** (**7**)	20.7 (6)	0.0 (0)	3.4 (1)
	Traditional doctors	F2 (408)	**35.5** **(145)**	**28.2** **(115)**	26.0 (106)	5.9 (24)	3.7 (15)	0.7 (3)
		F1 (316)	**30.4** (**96**)	**38.0** **(120)**	25.3 (80)	2.5 (8)	2.5 (8)	1.3 (4)

Results from F2 (2018) are presented first, followed by results from F1 (2017) beneath for comparison; in order to provide a sense of the distribution of answers, the two most common responses from each doctor group are shown in bold

F1, Foundation Year 1; F2, Foundation Year 2; LIFT, Longitudinal Integrated Foundation Training

#### Doctors’ perception of support received and their ability to cope

A greater percentage of LIFT doctors considered themselves to have been well, very well or extremely well supported in terms of both pastoral/personal and educational support *versus* traditional doctors (Table 2).

**Table 2:** Proportion of doctors who felt well supported and had the perceived ability to cope across F1 and F2

	Year	LIFT doctors, % (n)	Traditional doctors, % (n)
Total doctors	F2 F1	(12) (29)	(408) (316)
Pastoral/personal support^a^	F2	83.3 (10)	71.3 (291)
	F1	86.2 (25)	63.3 (200)
Educational support^a^	F2	100.0 (12)	72.8 (297)
	F1	79.3 (23)	67.1 (212)
Doctors rating their ability to cope as ≥ 7	F2	83.3 (10)	87.2 (356)
	F1	86.2 (25)	84.8 (268)
Supervisors rating the doctor’s ability to cope as ≥ 7	F2	83.3 (10)	82.4 (336)
	F1	55.2 (16)	86.7 (274)

Results from F2 (2018) are presented first, followed by results from F1 (2017) beneath for comparison

^a^Doctors considering themselves to be well, very well or extremely well supported

F1, Foundation Year 1; F2, Foundation Year 2; LIFT, Longitudinal Integrated Foundation Training

On average, doctors’ perceived ability to cope remained similar for both LIFT and traditional doctors and was consistent over time. However, when the distribution of ratings was considered, there was a marked increase in the percentage of LIFT supervisors who rated their doctors’ ability to cope as high (i.e. ≥7 out of 10) during F2, increasing from 55.2% to 83.3%.

#### Career intentions of doctors

Significantly more LIFT doctors (9.6% more, p < 0.05) intended to become general practitioners than traditional doctors (23.1% *versus* 13.5%, respectively; Table 3). Moreover, at the end of foundation training, significantly more LIFT doctors rated the extent to which they felt well informed and able to consider a GP career rather than a hospital career as high (91.7% *versus* 72.3%, respectively; p < 0.05). Similar proportions of LIFT (53.8%) and traditional (52.2%) doctors reported not immediately going into UK specialty training at the end of their foundation training.

**Table 3:** Career intentions of LIFT and traditional doctors

	LIFT doctors, % (n)	Traditional doctors, % (n)
Total doctors	(13)	(406)
Intentions upon completion of foundation training		
GP	23.1 (3)	13.5 (55)
Hospital	23.1 (3)	34.2 (139)
Not into UK specialty training	53.8 (7)	52.2 (212)
Total doctors	(13)	(406)
Intentions at the start of foundation training		
GP	30.8 (4)	16.7 (68)
Hospital	53.8 (7)	52.2 (212)
Not into UK specialty training	15.4 (2)	31.0 (126)
Total doctors	(12)	(408)
Doctors who felt well informed and able to consider a GP *versus* hospital career^a^	91.7 (11)	72.3 (295)

^a^Percentage of doctors rating themselves as high

GP, general practice; LIFT, Longitudinal Integrated Foundation Training; UK, United Kingdom

### LIFT doctor focus group assessment

In F1, 41 (87.2%) LIFT doctors participated; in F2, 27 LIFT doctors (57.4%) agreed to do so with a mean of 3 LIFT doctors participating across all eight sites. The results presented here encompass four themes that emerged from the focus groups which relate to doctor experiences in on-call secondary care exposure, communication skills and education in GP, relationships within the care setting, and the structure and implementation of LIFT.

#### On-call and secondary care exposure

In secondary care and the acute setting, doctors spoke of how participating in on-call activities during F2 had improved their confidence and self-perceived clinical competence:

“We’ve got on calls this year in every block which has been a massive thing for me feeling more competent.”

“I think I got more out of one on call shift than I probably did out of a week in hours, in terms of learning experiences… I’ve definitely felt more confident in managing acute unwell patients, taking responsibility for that.”

“...for my medical job I was apprehensive but after you’ve done a couple of on calls in medicine you just catch up.”

Additionally, some doctors expressed that they had coped better in the secondary care setting due to GP time providing a break from the hospital and acute patient cases; however, for some there remained the issue that over the duration of their foundation training they had received less on-call experience than traditional doctors and some viewed their reduced exposure to secondary care (owing to time out in GP) as a problem and spoke of the impact upon their abilities and confidence:

“It’s just that self-doubt that you’re not as good as everyone else, that’s been a really big thing, particularly this year, I think it’s been a lot worse. I think I felt about the same as everyone else at the end of F1 because I was doing those on calls still, so I had exposure, but this year we’ve hardly done any on calls.”

“I think if I compare myself to any of the other F2s who haven’t been on LIFT I think I will be inferior to them and I don’t genuinely think that’s a confidence thing, I think it’s my abilities, I have less experience on the wards than they have.”

#### Communication skills and educational experience in GP

Doctors’ patient communication skills were enhanced and there were increased opportunities to develop independent thinking as well as developing the ability to take effective patient medical histories, skills benefiting both primary and secondary care environments:

“In hospital… you don’t get good training on how to talk to people, so automatically if it’s not medical you just put them aside, you don’t even talk to them, whereas in GP you’re kind of forced to have the communication skills because you need to talk to these patients. So automatically your communication skills are a lot better and you talk more to people. In hospital medicine if you do it all the time, you have that mentality that I want to get my jobs done, it becomes just very tick box, you don’t really communicate unless you have to and you don’t have to, so you don’t communicate. So in a way overall I think it’s (LIFT) a really good thing to do. I’m actually happy that we did it.”

“...my communication skills are maybe better than they would have been, my consultation skills are probably better in hospital… and I do feel like I give a lot more to the patients than my seniors do, just by the way I’m asking questions, so from that perspective I’ve improved.”

“I would also like to say that I think some of our clinical skills are better in terms of other FY1s and FY2s because of GP, so doing specular exams and obs & gynae and paediatrics and that kind of thing I think we are a lot better than other doctors at our level, because we’ve seen them since the beginning… So, I suppose in some way it’s been good the GP aspect in terms of clinical skills and clinical knowledge.”

The continual relationship with their GP supervisor throughout their foundation training was also appreciated and was of great benefit in terms of receiving educational and clinical advice:

“At ours we had the opportunity to have watched clinics sometimes as well so you get immediate feedback from your supervisor, which I think if you weren’t on LIFT it would be difficult to get that sort of one on one supervision.”

“I think one of the positives (of LIFT) was to have a supervisor… building a relationship with them, they get to tailor to your needs and what you need to learn because they get to know you quite well and they can see where you lack experience and knowledge.”

“…it’s a definite, definite benefit having the same supervisor for two years rather than changing every four months. I can’t imagine feeling anything like as supported if I’d not had the same supervisor for two years.”

#### Relationships within the care setting

Doctors on the whole talked very positively about their relationships with the staff at their GP and expressed feeling more valued as part of their GP than hospital teams and being included at every level:

“GP has just got better and better really we’ve been there so long, I feel very much part of the team right from the receptionists, secretaries, everyone is absolutely brilliant. They’ve supported me in every way, they don’t see me as any different, they don’t go ‘oh, you’re part-time’, you’re one of our team and involved in everything from emails that go out about new guidelines to the team coffee rota. I am part of their team not just a junior doctor who floats in and out on a placement; they really, really value me so I feel really well supported there.”

“I find it very, very hard to build relationships in hospital and even still now you’ll see my F2 colleagues in the medical environment and they’ll be just ‘hi how are you doing? To registrars and consultants and all sorts, but I still feel like I don’t know anyone really.”

Doctors also reported being able to see their GP patients more holistically and as part of a family and, therefore, were better able to appreciate the impact of health both upon a patient and their relatives:

“…that sort of social side of things (in GP) makes you more aware of the process that people go through… I think you become aware of the whole story and actual learning wise… I think you can see the sort of process that they have to go through.”

“I’ve loved seeing the Mum, the Grandma, the child, I’ve loved seeing the sister bring in their kids and then seeing the other sister for something else and just piecing together the family. I’m recognising why they are a certain way and why I have to communicate in a different way. It’s definitely given me an opportunity to do that. I’ve mainly actually enjoyed GP a little bit more because they feel like more than just patients, they’re part of the practice and the family is there, the whole family comes to you so you feel part of the community.”

However, the issue remained that many doctors were unable to follow patients along the pathway between primary and secondary care as LIFT had intended:

“I found it very disruptive last year because of the ward jobs we had and that continuity that you need on a day to day ward job, you just didn’t have, so you’d get demoted to doing stuff and you didn’t learn from, so you’d be doing bloods, TTOs (‘To-Take-Outs’), you weren’t allowed to see anybody on the ward round because ‘there’s no point because you’re not here tomorrow’ kind of attitude.”

Due to the LIFT doctors’ absence to attend their designated GP training for part of their week, they sometimes found it difficult to build strong relationships with other members of their hospital teams as they rotated. Colleagues had negative perceptions of LIFT doctors’ absence in GP and their departures from the ward were largely felt to have had a negative impact:

“…people have confused LIFT with less than full time which has just been a nightmare from start to finish, people being like ‘why are you not full time?’ and having to be always explaining, explaining… Even now some 18 months on someone e-mailed me asking why I wasn’t full time.”

“…on surgery placement the surgeons were very much like ‘oh you’re on that LIFT job, you’re not here all week, oh it’s going to be negative for you’… and I think in a way I did feel like the one who was there part time. They much more regarded the full time person as a very good doctor and they are judging me too quickly because I’m the one who’s not there all week because actually I might have some attributes that are not being quite seen because of this cloud in the way which is LIFT, being a LIFT doctor.”

#### Structure and implementation of LIFT

For some LIFT doctors, continuity with their GP supervisor had a positive impact and the mix of GP and hospital had helped to avoid burnout. However, there was also strong evidence that doctors working three sessions within GP remained an issue, due to the stress and disruption caused by the half-day. As doctors have to attend both of their workplaces in one day, travel, handovers and leaving/arriving on time at the afternoon session all continued to be problematic. Doctors discussed missing team meetings and struggling with patient continuity due to their absence from each workplace on some days of the week:

“…this half a day business, honestly it blows my mind. I really don’t, I have anxiety, I feel sick on these days… I physically have to make sure I finish on time, and for me, I know I’m on ED and if it says 1.00 I have to be here for 1.00 so that stresses me out the whole morning, I’ve got to be changed here and ready and I just feel like what is the point of this half day? Like it’s just logistically so stressful. It’s so disruptive… The demand of the work is just too much in that half… if you’re on a medical ward you’re just chasing your tail, you don’t know what you’re doing. It’s just damage control; it’s not really doing anything properly.”

“It’s just a shame, because the idea of LIFT is actually good, I do support it and if I could design LIFT I could design a great LIFT programme, but it wasn’t designed well, which is a shame because I do feel that at the end of our F2 we’re going to be really good doctors.”

## Discussion

Implementation of the LIFT programme aimed to enhance the quality of the foundation training experience and provide doctors with more experience in the primary care setting [3]. The results presented here suggest that the quality of training was enhanced in specific areas, just as similar longitudinal programmes have benefitted individuals in other medical training systems [12]. Doctors performed better in terms of the General Medical Council’s six domains for good medical practice in line with the Department of Health’s mandate in 2015 [14]. Importantly, the positive outcomes from the LIFT programme are also aligned with the current Department of Health and Social Care mandate in 2022 [12, 15], which aims to deliver improved healthcare, in part, through better-quality education and training of doctors.

The LIFT programme enabled development of longitudinal patient relationships, which resulted in greater understanding of the patient’s perspective of the care they receive and contributed to doctors’ job satisfaction. Furthermore, the programme also enabled doctors to have a greater understanding of how primary and secondary care work together in the NHS and improved their resilience, stability in work and training, work-life balance and ability to make informed career choices, as well as be better supported. Moreover, similar to results from global longitudinal programmes [10], our analysis shows that more LIFT doctors felt better informed to consider a GP career than their traditional peers and intended to become general practitioners. It should be noted that a greater number of LIFT doctors than traditional doctors had already intended to pursue a GP career before embarking on foundation training (30.8% *versus* 16.7%, respectively); however, due to the meritocratic algorithm employed to match foundation posts, this is unlikely to have impacted the fact that LIFT doctors felt better informed to make this career choice.

LIFT emerged as increasingly positive in the 2018 survey, possibly due to the programme having become more established and initial set-up issues having been resolved, as deduced from comments made by survey participants. In comparison to the 2017 survey, there generally appeared to be less disparity between the opinions of doctors and their supervisors, presumably due to their relationships having further developed over the course of F2 and the deeper understanding of each other. The lower response rates in 2018 from LIFT doctors and their supervisors may reflect more contentment with the scheme, hence them feeling less motivated to respond to the survey as a way of airing concerns.

There were certain limitations to the LIFT pilot scheme which provide valuable learnings and also support the need for practical adaptations to the programme. One of the key objectives of the LIFT programme is that doctors have the opportunity to follow up with a range of patients as their illness is managed across both primary and secondary care settings. This was challenging to arrange in some cases and thought needs to be given on how to effectively manage this in the future in order to enhance doctors’ learning experiences. The reduced exposure to secondary care resulted in doctors reporting a lack of confidence in the hospital setting with a feeling that traditional doctors had better working relationships with the hospital teams. Therefore, particular consideration needs to be given to supporting doctor confidence during out-of-hours duties in the hospital setting. Working for a half-day in GP was disruptive for some LIFT doctors particularly with regards to travelling between sites; consequently, working only whole days as opposed to half-days in GP may support resolution of these concerns. Certain hospital specialties also found accommodating doctors’ absence challenging and consideration is needed for supporting specialties who have less experience of hosting less than full-time doctors. Some Trusts felt it easier not to include LIFT doctors on the out-of-hours rota due to the increased complexity of releasing them to their GP placement each week. While this had a detrimental effect on doctors’ pay, it was something that was addressed during the LIFT programme to give equal opportunity to the LIFT doctors for undertaking out of hours work compared with traditional doctors in the same specialty. It should be noted that, as awareness of the LIFT programme increased, there was a greater understanding within the hospital teams of when and why LIFT doctors were, at times, absent from the hospital and attitudes towards them changed as a result; mindfulness in doctor handover processes proved useful in these circumstances and an increased understanding and awareness meant that some sites made local changes to the LIFT schedule, e.g. rota amendments, more readily. Our results confirmed that stress management and help to achieve an improved work-life balance are still problems, and particularly needed by traditional doctors. As such, some LIFT and traditional doctors may still benefit from an increased provision of educational/pastoral support and information to enable them to cope with the issues they have experienced during their foundation training. While not within the scope of this paper, the authors note that another limitation of the LIFT programme was the uncertainty and anxiety some LIFT supervisors, their colleagues and administrative staff felt when supporting LIFT doctors. Consequently, increased provision of support for supervisors involved in the new scheme should be considered to enhance confidence in their role as a LIFT supervisor. Despite the limitations highlighted above, it is important to note that most negative perceptions or challenges experienced by doctors at the start of LIFT were generally ameliorated by the end of the 2-year programme.

It is also worth noting that, despite this pilot scheme consisting of a small number of LIFT doctors, the benefits emanating from the programme has seen its implementation expand since 2018. Indeed, the LIFT programme has been expanded and adapted across the East of England, North West, Wales and Yorkshire foundation schools, and has explored the recruitment of Physician Associates and different specialties such as psychiatry instead of GP.

## Conclusions

The results presented herein suggest that the traditional FP has the capability to evolve and have aspects improved in order to enhance doctor experiences and competencies with a view to increasing standards of healthcare. The LIFT programme has the potential to enhance foundation training in the UK with earlier insights into specialties such as GP being especially useful to help inform career choices. Continued assessment of both the advantages and disadvantages of the LIFT programme has the potential to further improve its implementation and, in time, LIFT may help to engage and retain doctors who are more compassionate and who exhibit a greater patient-centred and holistic approach for enhanced delivery of care.

## Data Availability

The datasets used and/or analysed during the current study are available from the corresponding author on reasonable request.

## Abbreviations

DME: Director of Medical Education
FP: Foundation programme
F1: Foundation Year 1
F2: Foundation Year 2
GP: General practice
LIFT: Longitudinal Integrated Foundation Training
NHS: National Health Service
NWoEFS: North West of England Foundation School
UK: United Kingdom
